# Determination of the causes and the effects of storage conditions on the quality of silo stored wheat (*Triticum aestivum*) in Zimbabwe

**DOI:** 10.1007/s13659-012-0004-5

**Published:** 2012-02-19

**Authors:** Tinotenda Admire Mhiko

**Affiliations:** Peoples’ Friendship University of Russia, 6 Miklukho-Maklay Street, Moscow, 117198 Russia

**Keywords:** *Triticum aestivum*, falling number, aflatoxins, silo

## Abstract

There are still cases of millers returning poor quality red wheat to the Zimbabwe Grain Marketing Board (GMB) and this has been an ongoing problem over the past few years. A larger amount of this wheat has discoloured and damaged embryos and it is discounted by millers because the germs are brittle and they crumble easily. There have been also many rejections of the red wheat particularly by major traders. Therefore there was an urgent need to investigate the causes and effects of storage conditions on the quality of silo-stored red wheat, since red wheat is one of human beings’ main food supplies. A representative sample of 2.25 kg of red winter wheat was randomly collected from the common red winter wheat incoming to the Grain Marketing Board Depot for storage. This representative sample of 2.25 kg was used as the control sample and its test density was determined. The control sample was then finely ground and analysed for protein, moisture, ash, aflatoxins and falling number. The red winter wheat was then stored in six different silos for a period of 5 months, with each silo having different humidity and temperature conditions. Representative samples of 4.5 kg were randomly collected monthly from each silo during the storage period. The test densities of the representative samples were determined. These representative samples were then finely ground and analysed for protein, moisture, ash, aflatoxins, and falling number. The results of the red wheat in storage were then compared with those of the control sample and analysed by analysis of variance (ANOVA) at the 5% level of significance. Results obtained after data analysis suggest that there were significant differences in the protein content, moisture content and falling number of the wheat before and after storage. However, differences in test density, aflatoxin and ash contents of the wheat before and after storage were not statistically significant at the 5% level of significance. The deterioration in wheat quality was attributed to the high storage temperature and humidity conditions. It was also concluded that the optimum conditions for wheat storage are a temperature of 15 oC and a humidity of 60%.

## Introduction

Red wheat is one of human beings' main food supplies. The research focused on investigating the causes and effects of storage conditions of temperature and humidity on the quality of silo stored red wheat with time. Despite the research being conducted in 2006, the GMB is still employing the same storage conditions and the organisation have not managed to improvise on the storage conditions due to the tough economic sanctions on Zimbabwe which have also resulted in retrenchment of some of the employees at the Grain Marketing Board.

There are still cases of millers returning poor quality red wheat to the Zimbabwe Grain Marketing Board and this has been an ongoing problem over the past few years. A larger amount of this wheat has discoloured and damaged embryos. Such wheat is discounted by millers because the germs are brittle and they crumble easily. In January 2006, there were many rejections of wheat particularly by major traders (personal observation, 2006). A total of 56 loads were returned and of the 56 loads returned, there were 102 complaints, 48 complaints were for test density, 46 were for discolouration and 8 were for insects.

The Food and Agriculture Organization (FAO) estimates that 25% of the world food crops are affected by aflatoxins before and during storage. Foods and feeds contaminated by aflatoxin can cause reduced growth rates, immune suppression and death in human beings and animals. There have been also many reports on the formation of toxic compounds in mould damaged wheat and food by the FAO food security mission. The FAO food security mission also noted that the effects of storage conditions such as fungi caused considerable loss, when grain was stored for 3 months or longer. Regulatory concerns also mandate that wheat should meet the established quality parameters before and after storage. Thus, an investigation on the impact of storage conditions on the quality of wheat during storage is of prime importance.

Wheat contributes 10–20% of the daily calorie intake of

*To whom correspondence should be addressed. E-mail: tinomhiko@yahoo.co.uk people in over 60 countries.[1] There are more than a thousand varieties of bread prepared worldwide. Except for the warm tropics, wheat adapts to all diverse climatic conditions prevailing in agricultural lands and therefore it is harvested in the world all year round. Its wide adaptation to diverse environmental conditions, along with its unique characteristics of possessing a viscoelastic protein complex called gluten, are the main factors making wheat one of the most important food crops in the world. Wheat contains appreciable amounts of calcium and iron but the value of these minerals is partly discounted by the phytic acid and phosphate in the bran that interfere with their absorption.[2]

The principal storage conditions which affect the quality of wheat grain in storage are temperature and relative humidity. The interaction of these storage conditions equally impacts on the quality of wheat. The physical and chemical characteristics of wheat have a strong influence on its end use. Consequently wheat is commonly subject to grade criteria not necessarily applied to other grains such as test weight, protein content and falling number. The wheat grain is graded using the grain acceptance standards.

The quality of wheat flour is determined in most cases by quality of wheat grains. Wheat quality is determined by both environmental and genetic factors. The environmental factors include soil condition, crop management, storage conditions and weather throughout the growing season. How genetic factors affect wheat quality is determined by wheat breeders who develop new wheat varieties.

## Results and Discussion

**Protein Content of Wheat During Storage.** The protein content of wheat in all the silos decreased during storage regardless of storage locations. Statistical analysis of the above results conducted using ANOVA at the 5% level of significance showed that the protein content of the wheat decreased significantly ($p \leq 0.05$) during storage.

**Aflatoxin Content of Wheat During Storage.** The aflatoxin content of wheat in all the silos decreased during storage regardless of storage locations. Statistical analysis of the above results conducted using ANOVA at the 5% level of significance, showed that there was no significant decrease in the aflatoxin content of the wheat during the storage period.

**Moisture Content of Wheat During Storage.** Increases in moisture content of wheat during storage were noted in silos number 18, number 19 and number 20. Decreases were however noted in silos number 15, number 16 and number 17. Statistical analysis of the above results conducted using ANOVA at the 5 % level of significance showed that the moisture content of the wheat decreased significantly ($p \leq 0.05$) during the storage period.

**Falling Number of Wheat During Storage.** The falling number of wheat increased during storage in silos number 16, number 19 and number 20. A decrease was however noted in silos number 15, number 18 and number 17. Statistical analysis of the above results conducted using ANOVA at the 5 % level of significance showed that the falling number of the wheat decreased significantly ($p \leq 0.05$) during the storage period.

**Ash Content of Wheat During Storage.** The ash content of silo number 16 remained constant during storage. However, slight decreases were observed in the remaining silos. Statistical analysis of the above results conducted using ANOVA at the 5 % level of significance, showed that there was no a significant decrease of the ash content of the wheat during the storage period.

**Test Density of Wheat During Storage.** The test density of wheat in silo number 16 remained constant during storage. However, slight decreases in test density were observed in the remaining silos. Statistical analysis of the above results conducted using ANOVA at the 5% level of significance, showed that there was no significant decrease of the test density of the wheat during the storage period. The principal sources of losses in the wheat grain quality and quantity during storage were high temperature and high humidity conditions, moulds and insects (*Sitophilus granarius*), as well as their interaction during the storage period.

## Changes in Qualitative Properties

**Protein Content.** The protein content of the wheat before storage was 12.6 % (Table 1). However, the protein content of all the samples decreased with time regardless of the storage locations (Figure 1). The highest rate of decrease of protein content of the stored wheat was observed in silo number 15 where the protein content decreased from 12.6% to 10.8% during the storage period (Figure 1). The highest protein content deterioration rate of the wheat in this silo can be attributed to higher temperature and humidity conditions relative to the rest of the other silos. The proteolytic activity of the stored wheat might have been increased by higher temperature and moisture conditions in this silo. The endopeptidases cleave peptide bonds within the chain whereas the exopeptidases cleave single amino acids from either the carbon or nitrogen end of the molecule.[2] Both the endopeptidases and the exopeptidases break the protein polypeptide bonds into simple peptide chains. This might have decreased the protein content of the wheat during storage.

**Table 1. Qualitative and quantitative properties of the wheat before storage (control sample)**

| variable | value |
|---|---|
| protein | 12.6% |
| falling number | 298 s |
| aflatoxin content | 6.8 ppb |
| moisture content | 11.4% |
| ash content | 1.42% |
| test density | 82% |
| insect and pest infestation | Nil |
| discolourations and caking | Nil |

The lowest rate of decrease was noted in silo number 16, where the protein content of the wheat decreased from 12.6% to 12.5% (Figure 1). The lowest protein content deterioration rate of the wheat in this silo can be attributed to the lower humidity and temperature conditions. At lower temperatures, the activity of the peptidases is slow. Consequently, the rate of protein degradation of the stored wheat is also minimal.

Silo number 17 and silo number 20 had higher deterioration rates of protein relative to silo number 18 and silo number 19. This can also be attributed to higher temperature and moisture conditions in these silos. At high temperatures, the metabolism of protein is high.

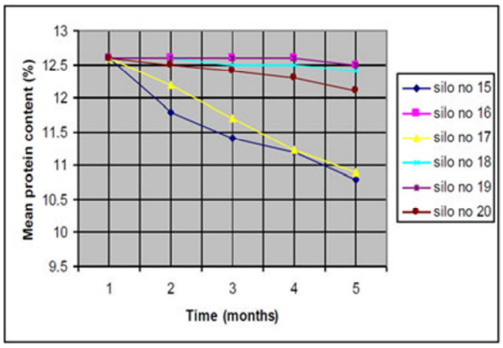

**Figure 1.** Mean variations in the protein content of wheat during storage

The decrease in protein content can also be attributed to the increased mould content of the wheat. The moulds produce mycotoxins which in turn, produce aflatoxins. Aflatoxins utilise nutrients in the wheat including proteins, for their growth and survival. This might have caused the decrease in protein content of the wheat during storage. The differences in the protein contents of the wheat before and after storage were statistically significant at the 5 % level of significance, as shown by the P values (Table 5).

**Table 5. Statistical analyses of the qualitative and quantitative properties**

| variable | F value | P value |
|---|---|---|
| protein | 2.74 | 3.61 |
| moisture | 2.74 | 3.71 |
| falling number | 2.74 | 3.98 |
| aflatoxin | 2.74 | 1.74 |
| ash content | 2.74 | 0.12 |
| test density | 2.74 | 1.58 |

**Falling Number.** The falling number of the wheat before storage was 298 s (Table 1). Decreases in falling number of the stored wheat were however observed in silos number 15, number 17 and number 18 (Figure 4). The decrease was more pronounced in samples from silo number 15 in which the falling number decreased from 298 s to 288 s during the storage period (Figure 4). The decrease in falling number was higher in silo number 17 (298 s to 290 s) relative to silo number 18 (298 s to 292 s) (Figure 4). The decrease in falling number suggests that the alpha-amylase activity increased over the storage period.

This can be attributed to the pre-germination process that might have occurred due to increased moisture contents of the wheat in silos number 15, 17 and 18. High moisture conditions cause germination of the wheat kernels. For germination to occur energy is required and this energy is obtained from simple sugars. Consequently, the alpha-amylases rapidly hydrolyse the starch in the endosperm of the wheat kernels, forming fragments of glucose sub-units called maltodextrins. The maltodextrins are then hydrolysed by maltase into glucose. Falling number values bear a complex inverse relationship with the quantity of alpha-amylase in the sample.[3]

The samples from silos number 15, number 17 and number 18 showed a greater decrease in falling number (Figure 4). This is because the moisture content might have been optimum for alpha-amylase activity.

Increases in falling numbers with time were observed in wheat samples from silos number 16 (298 s to 308 s), number 19 (298 s to 314 s) and number 20 (298 s to 312 s), (Figure 4). The increase in falling numbers also suggests that the alpha-amylase activity decreased over the storage period. This can be attributed to the increased fungal and mould content of the wheat during storage. The moulds utilise nutrients in the germ and this kills the embryo of the wheat kernels. This results in a decrease in germinative energy of the wheat, hence alpha-amylase activity is also reduced. This might have decreased the falling number of the wheat during the storage period.

The differences in the falling numbers of the wheat before and after storage were statistically significant at the 5 % level of significance, as shown by the P values (Table 5).

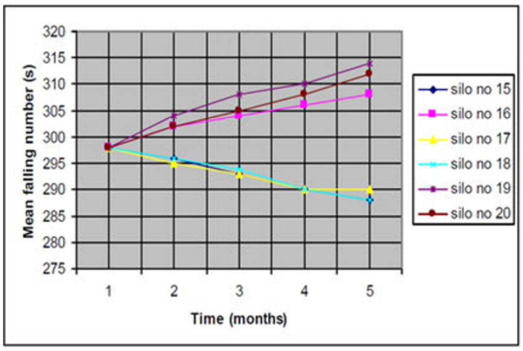

**Figure 4.** Mean variations in the falling number of wheat during storage

**Aflatoxin Content.** The initial aflatoxin content detected in the wheat before storage was 6.8 parts per billion (ppb) (Table 1). This suggests that the wheat was already infected when it was stored. This might have been caused by field fungi. Field fungi invade the grain before harvest while the crop is still in the field and can affect the appearance quality and appearance of the wheat seed or grain.[1] Invasion by field fungi may be more severe if the crop has been damaged by insects, birds or hail. Field fungi result in the development of mycotoxins which in turn develop into aflatoxins.[4]

Both the aflatoxin B1 and aflatoxin G1 were identified in all the silos (Table 4). The highest rate of increase of levels of aflatoxins was noted in silo number 15 (Figure 2) and this can be attributed to high temperature and humidity conditions in these silos. Aflatoxins grow rapidly within the temperature range of 20 °C to 44 °C.[4] The optimal temperature range for the growth of important grain moulds is between 25 °C and 30 °C[5], which is close to the temperature range employed in silo number 15 (20 °C to 40 °C). This might have accelerated the development of several species of Aspergillus and Penicillium, which tend to produce aflatoxins.

The increase in aflatoxin content can also be ascribed to the inoculation of fungi on the stored wheat by *Sitophillus granarius*. Insects can inoculate fungi on stored grain.[6] The relative abundance of *S. granarius* was highest in silo number 15 (Table 4) and this might also have accelerated the development of aflatoxins in this silo.

**Table 4. Aflatoxin types identified in the silos during the storage period**

| period | silo number 15 | silo number 16 | silo number 17 | silo number 18 | silo number 19 | silo number 20 |
|---|---|---|---|---|---|---|
| 7-Feb | B1 | B1 | B1 | B1 | B1 | B1 |
| 7-Mar | B1, G1 | B1 | B1 | B1 | B1 | B1 |
| 7-Apr | B1, G1 | B1 | B1, G1 | B1, G1 | B1 | B1, G1 |
| 7-May | B1, G1 | B1 | B1, G1 | B1, G1 | B1, G1 | B1, G1 |
| 7-Jun | B1, G1 | B1, G1 | B1, G1 | B1, G1 | B1, G1 | B1, G1 |

The slowest rate of aflatoxin development was noted in silo number 16 (Figure 2). This can be attributed to the low temperature and moisture conditions in this silo, which do not favour the growth of aflatoxins. Fungi or moulds or aflatoxins grow very slowly at lower temperatures (5 °C to 16 °C).[7]

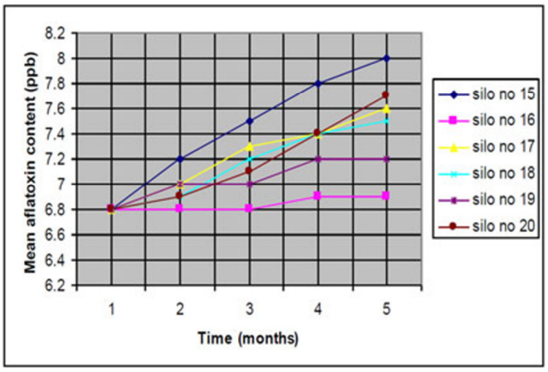

**Figure 2.** Mean variations in the aflatoxin content of wheat during storage

The respiration of both the stored wheat and *Sitophilus granarius* can also be ascribed to the increase in aflatoxin development. Respiration produces $CO_2$, heat and $H_2O$ which in turn favour growth of aflatoxins. Respiration also causes the oxidation of food materials to supply energy forbiochemical processes.[8] However, the differences in the aflatoxin contents of the wheat before and after storage were statistically significant at the 5 % level of significance, as shown by the P values (Table 5).

**Ash Content.** The initial ash content detected in the wheat before storage was 1.42 % (Table 1). The ash content remained constant in silo number 16 throughout the storage period (Figure 5). However a slight decrease was noted in all the remaining silos. The rate of decrease of ash content was highest in silo number 15 (1.42 % to 1.39 %), (Figure 5). The slight decrease in ash content can be attributed to respiration of the wheat during storage. During respiration, there is a decrease in the levels of carbohydrates, hydrogen and oxygen. Respiratory losses of the carbon cause weight losses in the product, hence the ash content subsequently decreases. Respiration also decreases the total food value (energy content).[9] However the differences in the ash contents of the wheat before and after storage were not statistically significant at the 5 % level of significance, as shown by the P values (Table 5).

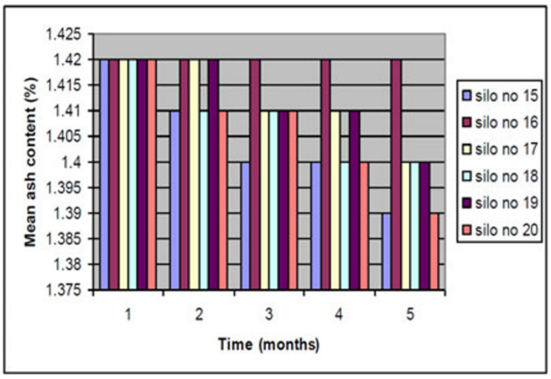

**Figure 5.** Mean variations in the ash content of wheat during storage

**Insect Infestation.** Presence of the rusty grain weevil *Sitophilus granaries* was also noted in the wheat samples from silos number 15, 17, 18, 19 and 20 (Table 2) and the insect is shown in Figure 7. The weevil feed on the germ (embryo) part of the whole wheat kernel. The highest concentration of *S. granarius* was noted in silo number 15, this silo had a relative abundance of 28%, (Table 3). This can be attributed to high temperature conditions in this silo, which favours growth of the weevil. However, no any form of infestation was noted in silo number 16 and this can be attributed to the low temperature conditions in this silo which inhibits growth of *S. granarius*. High temperatures of 28 °C–30 °C and relative humidity of 65%–80% favour microbial and insect growth.

**Table 2. Distribution of *Sitophilus granarius* in the silos during the storage period**

| period | silo number 15 | silo number 16 | silo number 17 | silo number 18 | silo number 19 | silo number 20 |
|---|---|---|---|---|---|---|
| 7-Feb | --- | --- | --- | --- | --- | --- |
| 7-Mar | --- | --- | --- | --- | --- | --- |
| 7-Apr | +++ | --- | --- | --- | --- | --- |
| 7-May | +++ *C* | --- | +++ | --- | --- | +++ |
| 7-Jun | +++ *C* | --- | +++ *C* | +++ | +++ | +++ *C* |

+ + + = Infestation with *Sitophilus granaries*; --- = No infestation; *C* = Caking

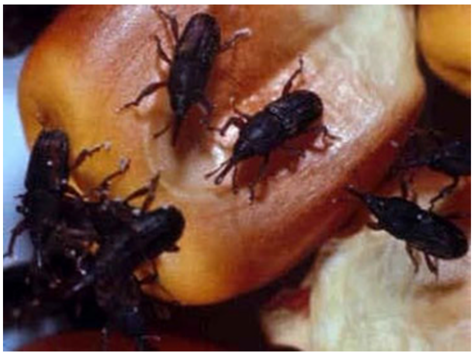

**Figure 7**. *Sitophilus granarius* (GMB, 2006)

The results obtained with regard to insect infestation suggest that infestation was also favoured by high moisture conditions of the wheat. At lower temperature and moisture conditions, the growth of insects is not favoured.[10]

**Table 3. Relative abundance (%) of *Sitophilus granarius* in the silos during the storage period**

| silo No. 15 | silo No. 16 | silo No. 17 | silo No. 18 | silo No. 19 | silo No. 20 |
|---|---|---|---|---|---|
| 28.0 | 0.0 | 20.2 | 11.6 | 14.6 | 25.6 |

**Moisture Content.** The initial moisture content detected in the wheat before storage was 11.4% (Table 1). A decrease in moisture content was observed in samples from silos number 15 (11.4% to 10.2%),17 (11.4% to 10.4%) and 18 (11.4% to 10.3%), (Figure 3). The results suggest that an increase in temperature had a corresponding decrease in moisture content of the wheat during the storage period. The decrease in the moisture content can be attributed to the increased temperatures. As the temperature increases, the difference between the partial pressure on the surface of the wheat grain and the vapour pressure in the ambient air becomes the driving force for vaporisation of moisture from the kernel surface to the air. This might have caused the rapid water loss from the stored wheat. Consequently, the moisture content of the wheat kernels is reduced, a process called desorption.[9]

Increases in moisture content of the wheat were however noted in wheat samples from silo number 19 (11.4% to 12.3%) and silo number 20 (11.4% to 12.1%). The increases in moisture content can be attributed to aerobic respiration of the stored wheat. Aerobic respiration results in complete oxidation of hexose and this yield $CO_2$, $H_2O$ energy.[11] Consequently, the loss in mass and an increase in moisture content of the grain occurs. A rise in the temperature of the grain also occurs and this in turn creates favourable conditions for wheat spoilage. The increasing moisture content might also have been caused by respiration of *Sitophillus granarius*. The water produced during respiration might have contributed to the increased moisture content of the stored wheat.

The increase in moisture content can also be ascribed to hot spots due to *Sitophillus granarius*. Since grain is a poor conductor of heat, the temperature in the hot spot increases. These hot spots cause the creation of temperature gradients within the silo. This in turn causes convection currents of moisture from warmer to cooler regions within the stored wheat and consequently formation of condensation. This might have also caused the increase in moisture content of the wheat during storage.

The increase in moisture content can also be attributed to the hygroscopic nature of all stored grain, that is, they absorb moisture from humid air and lose moisture to dry air until equilibrium is established. The higher the humidity is the greater the amount of moisture the grain can absorb and vice versa.[12]

Silo number 16 had the lowest rate of increase of moisture content with time (11.4% to 11.8%), (Figure 3). The slow rate of increase of moisture content in this silo might have been due to low humidity and temperature conditions throughout the storage period. The differences in the moisture contents of the wheat before and after storage were statistically significant at the 5% level of significance, as shown by the P values (Table 5).

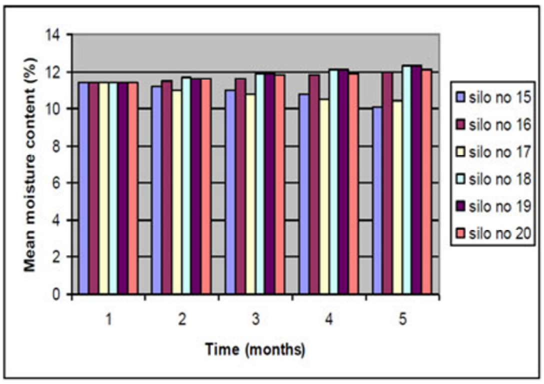

**Figure 3.** Mean variations in the moisture content of wheat during storage

**Germ Discolourations and Caking.** No any forms of discolourations were observed on the wheat before storage (Table 1). However, germ discolourations were later observed in samples from silos number 15 and number 20 (Table 3). The discolourations might have been caused by fungal deterioration of the wheat grain during storage. Fungal deterioration causes aesthetic changes including discolouration, caking and musty odours.[13]

Caking of the wheat during storage was also noted in samples from silos number 15 to number 20 (Table 2). Caking is a dynamic process that is dependent on the moisture content of the grain. However, the caking was only observed in samples from silo number 15. The caking can be attributed to both high moist and high temperature conditions of silos number 15 and number 20. The higher the moisture content is the faster the rate of caking.[14] Caking occurs due to the material surface plasticisation. Material surface plasticisation is the sticking together of wheat kernels due to high moist conditions.

### Changes in Quantitative Properties

**Test Density.** The test density remained constant in silo number 16 (82%) throughout the storage period. However, steady decreases were observed between March and June in all the remaining silos (Figure 6). The slight decreases decrease in test density can be attributed to the increased moisture content of the wheat during storage. The wheat with high moisture content undergoes an autolytical digestion (self-digestion). During autolytic digestion, breakdown of nutrients including carbohydrates to supply energy for biochemical processes takes place, this might have decreased the test density of the wheat during storage.

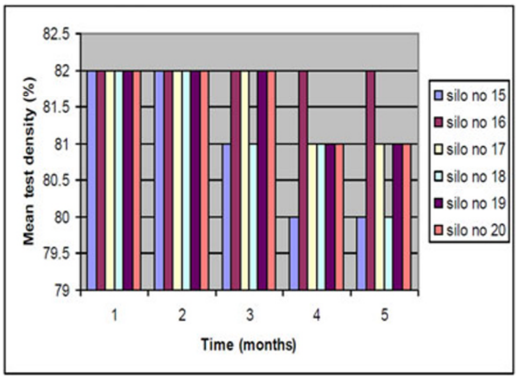

**Figure 6.** Mean variations in the test density of wheat during storage

The slight decrease in test density during the storage period can also be ascribed to aerobic respiration of the wheat grain during storage. The living cells of the stored wheat respire continuously utilising the starch substrate in the endosperm. The loss of the starch substrate from the stored might have caused the decrease in test density. For moisture contents above 13%, respiration causes losses in nutritive value and dry matter. However the differences in the test densities of the wheat before and after storage were not statistically significant at the 5% level of significance, as shown by the P values (Table 5).

## Experimental Section

The research was done using the common red winter wheat variety (*Triticum aestivum*). A representative sample of 2.25 kg of red winter wheat was randomly collected from the common red winter wheat incoming to the Grain Marketing Board Depot for storage. This representative sample of 2.25 kg was used as the control sample and its test density was determined. The control sample was then finely ground and analysed for protein, moisture, ash, aflatoxins and falling number. The wheat was then stored in six different silos for a period of 5 months, with each silo having different humidity and temperature conditions. The humidity and temperature of silo number 15 were both increased from 65% to 85% and 20 °C to 40 °C respectively, during storage. In silo number 16, both humidity and temperature were kept constant at 60% and 15 °C respectively throughout the storage period. In silo number 17, temperature was kept constant at 20 °C, while humidity was increased from 65% to 85%, during storage. In silo number 18, temperature was increased from 20 °C to 40 °C, while the humidity was kept constant at 65%, during storage. In silo number 19, both humidity and temperature were decreased from 65% to 45% and 40 °C to 20 °C respectively, during storage. In silo number 20, both humidity and temperature were kept constant at 75% and 22 °C respectively, throughout the storage period. The storage conditions in silo number 20 are currently employed by GMB for wheat storage. Temperature and humidity of the silos were thermostatically controlled using automatic aeration controllers. Representative samples of 4.5 kg were randomly collected monthly from each silo during the storage period. The test densities of the representative samples were determined. These representative samples were then finely ground and analysed for protein, moisture, ash, aflatoxins, and falling number. The results of the red wheat in storage were then compared with those of the control sample and analysed by ANOVA at the 5% level of significance.

**Sample Preparation.** A double-tube spear was used to collect samples from the silos (Figure 8). A double-tube spear consists of two metal tubes, one fitting closely inside the other and each with several slots corresponding to similar slots in the other tube. The slots can open or close by turning the inner tube through 180 degrees.[15]

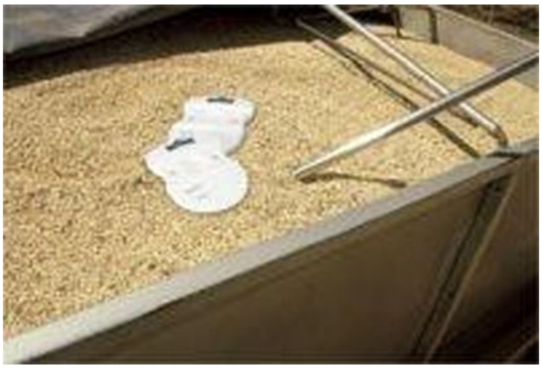

**Figure 8.** Double-tube spear for bulk wheat sampling (GMB, 2006)

### General Experimental Procedures

**Crude Protein Determination.** Crude protein content was determined using the Kjeldahl Tecator System.[16] One gram of milled flour sample was placed in a 500 ml Kjeldahl flask and 10 g of $Se_2O$ catalyst was dissolved in 30 ml sulphuric acid ($H_2SO_4$). The flask was heated on a mantle for 45 minutes in a fume hood whilst turning the flask at 15 minute intervals. The digest was then transferred quantitatively into a 250 ml volumetric flask and cooled on a running tap. The flask was then topped to the mark by adding distilled water. The digest (25 ml) was the pipetted into a husking distiller and 25 ml of 50% NaOH was added into the husking. The digest was steam distilled using the Tecator system and the ammonia produced was collected in a 100 ml conical flask containing 15 ml $H_2O_2$. The distillate was titrated with 0.01 $H_2SO_4$ and indicator (0.125 g methyl red plus 0.00825 g methylene blue) in 100 ml alcohol was used. The protein content of the calculated was as follows:

$$\text{crude protein} = \%\text{nitrogen} \times 6.25 \text{ (wheat flour)}$$

**Falling Number Determination.** Enzyme Falling number was determined according to GMB standard procedures[15], using the NIR Inframatic machine (model 1500), (Figure 9).

Falling number values bear a complex inverse relationship with the quantity of alpha-amylase in the sample. The ground wheat sample (7.00 ± 0.05 g), was weighed and transferred into a viscometer tube. Distilled water (25 ± 0.2 ml) was pipetted into the viscometer tube containing the ground wheat sample. A clean dry stopper was fitted into the top of a viscometer tube and an electrical shaker was then used to shake the sample vigorously so as to obtain a homogeneous suspension. The stopper was removed and a clean dry viscometer stirrer was used to scrape all solids which were adhering to the sides of the tube. The solids were scrapped into the suspension. The tube was replaced with a stirrer in the cassette. The cassette with the tube and stirrer was then placed into the groove in the cooling lid, within 40 ± 10 seconds after mixing. The stirrer tower was pulled forward, immediately after placing the tube in the boiling water bath and the test automatically started. When the test was complete, the falling number displayed on the digital display unit of the machine.

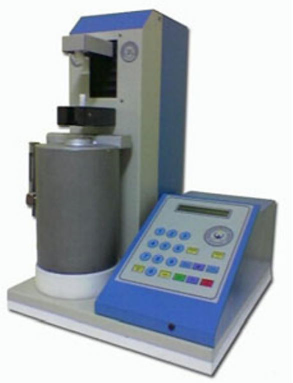

**Figure 9.** NIR Inframatic machine (model: 1500, GMB, 2006)

The method is based upon the rapid gelatinization of a suspension of flour or meal in a boiling water bath and the subsequent measurement of the liquefication by alpha-amylase of the starch contained in the sample.[2]

**Moisture Content Determination.** The moisture content was determined according to standard procedures[17] using the oven-dry method. An empty crucible was weighed and its mass was recorded as $M_0$. Then 5 g of the ground wheat sample was recorded as $M_1$. The crucible containing the 5 g ground wheat sample was placed in the oven and allowed to dry 200 °C for 2 hours. The sample was then removed from the oven and placed in a dessicator for 45 minutes. The sample was weighed after it had cooled and its mass was recorded as $M_2$. The sample was re-heated and at 15 minute intervals until a constant weight was obtained. The percentage moisture by mass was calculated as follows:

$$(\mathbf{M_1 - M_2/M_1 - M_0) \times 100}$$

Where:

$\mathbf{M_0}$ = mass (g) of the crucible

$\mathbf{M_1}$ = mass (g) of the crucible and the sample before drying

$\mathbf{M_2}$ = mass (g) of the crucible and the sample after drying

**Temperature and Humidity Control.** Temperature and humidity were controlled by means of by use of the automatic aeration controllers. Aeration cooling was achieved with airflow rates of 2 °C 3 litres per second per tonne delivered from fans driven by a 0.37 kilowatt (0.5 horsepower) electric motor for silos around 100 t.

**Aflatoxin Determination.** All Due to lack of resources, some aflatoxins were not analysed. Only the most potent carcinogens were tested for and these are aflatoxins B1 and G1. Those not analysed are aflatoxins B2, G2, M1, zearalenone, deoxynivalenol and ochratoxin A. The aflatoxin content was determined according to standard procedures.[18] Aflatoxins were tested using a 30 g wheat flour sample. The analysis consisted of 4 stages, namely extraction, clean up, purification, and quantification. During the extraction process, the wheat sample was mixed with 60 ml of distilled water and homogenized at 3000 revolutions per minute for 1 minute using a homogeniser to produce slurry. The 60 ml slurry was mixed with 60 ml of chloroform (an organic solvent) and shaken moderately for 2 minutes. The clean up process involved eluting the extracted slurry through a mini column packed with dextrose filters and allowing water (200 ml) containing 50 % ether solvent to run through the column. Purification of aflatoxins was conducted using thin layer chromatography (TLC). TLC produces full or complimentary information, enabling the mycotoxin to be unequivocally identified and if necessary quantified at the level of interest. The stationary phase in the chromatography was silica gel while the mobile phase was chloroform, a solvent. Quantification of aflatoxins was done by streaking 2 ml of purified whole wheat flour slurry onto ceramic ultraviolet (UV) plates and viewing under a UV light aflatoxin detector.

**Ash Content Determination.** The ash content was determined according to standard procedures.[18] The ground wheat sample (5 g) was weighed into a crucible and the organic matter was burnt off for 6 hours at 550 °C, using a muffle furnace. The sample was re-heated at 15 minute intervals until a constant weight was obtained. The sample was cooled in a dessicator and the amount of ash was determined by weighing.

**Test Density Determination.** The test weight was determined according to GMB grain acceptance standards.[15] The unground wheat sample (500 g) was poured into a hectoliter cup. The hectoliter cup was leveled to remove excess wheat grain. The weight of the wheat grain from the hectoliter cup was then measured on an electronic balance. The weight obtained is the test density of the sample.

**Data Analysis.** The results were analysed by ANOVA. The silos (each silo having different storage conditions of humidity and temperature) were treatments. The response variate were the changes in the qualitative properties of protein, moisture, falling number, aflatoxins, ash as well as the quantitative property of test density of the stored wheat over a period of 5 months.

**Acknowledgments**

I would like to express my sincere gratitude to the

Zimbabwe Grain Marketing Board for the support they gave me throughout the course of this research.

I would also like to thank my brother Mr. Philip Mhiko as well as my colleagues particularly Mr. Edson Nyambo whose great contribution made a positive difference towards the accomplishment of this piece of work. Their invaluable assistance made this research a success.

My final word of appreciation goes to all the lecturers from the Food Science and Post Harvest Technology Department at Chinhoyi University of Technology who taught me from 2004 to 2008.

**Open Access** This article is distributed under the terms of the Creative Commons Attribution License which permits any use, distribution, and reproduction in any medium, provided the original author(s) and source are credited.
